# Recognition and Treatment of Homozygous Familial Hypercholesterolemia by Primary Care Physicians: a Survey from the National Lipid Association

**DOI:** 10.1007/s11606-019-05620-4

**Published:** 2020-01-16

**Authors:** Linda Hemphill, Anne Goldberg, Kees Hovingh, Jerome Cohen, Dean G. Karalis

**Affiliations:** 1 Massachusetts General Hospital, Boston, MA USA; 2 Washington University School of Medicine, St. Louis, MO USA; 3 University of Amsterdam, Amsterdam, NL USA; 4 St. Louis University, St. Louis, MO USA; 5 Thomas Jefferson University Hospital, 227 North Broad Street, Suite 200, Philadelphia, PA USA

## INTRODUCTION

Homozygous familial hypercholesterolemia (HoFH) is an inherited disorder caused most commonly by mutations in the low-density lipoprotein (LDL) receptor gene. The very high levels of LDL-cholesterol from birth lead to early and widespread atherosclerosis, and guidelines recommend early and intensive lowering of LDL-cholesterol.^1^ The prevalence of HoFH in the general population is more common than previously estimated, with about 1 in 160,000 to 300,000 having HoFH. The National Lipid Association conducted a survey to evaluate how primary care and other clinicians diagnose and manage patients with HoFH. We focused on clinicians in primary care and general medicine since they are usually the first in the healthcare community to see these patients.

## METHODS

A total of 504 clinicians completed the survey across the USA from June to July 2018. The respondents had to be currently treating patients with elevated LDL-cholesterol and licensed to prescribe medications. Eligible medical disciplines included physicians, nurse practitioners, and physician assistants. Eligible specialty groups included family practice, general internal medicine, and cardiology.

## RESULTS

**Figure 1:**
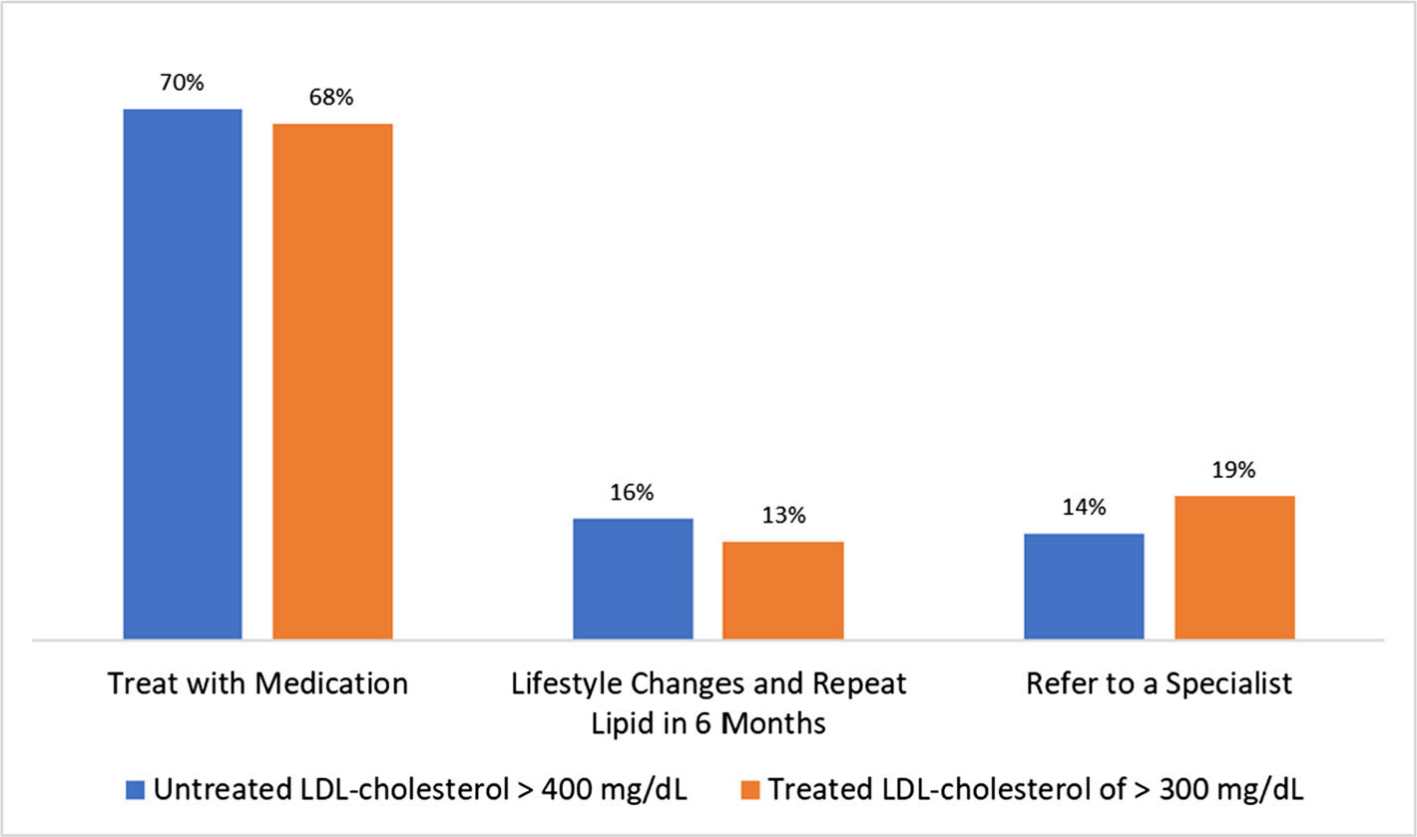
**Initial choices for what steps the survey respondents would take in treating patients with an untreated LDL-cholesterol** **>** **400 mg/dL and for patients with a treated LDL-cholesterol > 300 mg/dL.**

Of those who completed the survey, 85% were physicians, 99% were in primary care or general internal medicine, and 63% had access to a lipid specialist which varied by their location (36% urban, 30% suburban, and 13% rural). The answers to the survey questions are provided in Table 1 and Figure 1.

**Table 1:** Answers to the Survey Questions

Survey question	Answer	%
Do you have patients with an untreated LDL-C > or equal to 400 mg/dL?	Yes	69
	No	31
Do you have patients with a treated LDL-C > 300 mg/dL?	Yes	76
	No	24
What is your diagnosis for a patient with an untreated LDL-C > or equal to 400 mg/dL?	HoFH	54
	HeFH	24
	Mixed hyperlipidemia	16
	Familial Chylomicronemia	2
	High cholesterol due to poor lifestyle habits	5
What is your diagnosis for a patient with a treated LDL-C > 300 mg/dL?	HoFH	41
	HeFH	32
	Mixed hyperlipidemia	20
	Familial Chylomicronemia	2
	High cholesterol due to poor lifestyle habits	5
What method do you use to diagnose HoFH?	Genetic testing	37
	Clinical criteria	63
Does your practice have access to a lipid specialist?	Yes	63
	No	37
Does your practice have access to an LDL-apheresis center?	Yes	29
	No	71
In a patient with HoFH what risk factors would you use to determine ASCVD risk?	Level of LDL-C	87
	Level on non-HDL-C	53
	Coronary artery calcium scoring	66
	Level of Lp(a)	55
	Family history	90
Would you use a risk calculator to determine high risk for ASCVD in a patient with HoFH?	Yes	82
	No	18
What would you use to treat a patient with HoFH?	High dose statin	8
	Diet and exercise	63
	PCSK9 inhibitor	60
	Ezetimibe	39
	Fish oil	29
	Fibrate	17
	Bile acid sequestrant	15
	Niacin	15
	LDL-apheresis	13
	Low to moderate dose statin	11
	Plant sterols	7
	Lomitapide	7
What age would you start a male HoFH patient on LDL-C lowering medication?	< 18 (years old)	24
	> 18	76
What age would you start a female HoFH patient on LDL-C lowering medication?	< 18 (years old)	20
	> 18	80
What treatment goal would you use for an HoFH patient free from clinical ASCVD?	LDL-C decrease by at least 50%	33
	LDL-C < 70 mg/dL	24
	LDL-C < 100 mg/dL	43

*ASCVD*, atherosclerotic cardiovascular disease; *HeFH*, heterozygous familial hypercholesterolemia; *HoFH*, homozygous familial hypercholesterolemia; *Lp(a)*, lipoprotein (a); *LDL-C*, low-density lipoprotein cholesterol; *non-HDL-C*, non-high-density lipoprotein cholesterol; *PCSK9*, proprotein convertase subtilisin/kexin type 9

## DISCUSSION

Although most of our survey respondents manage patients with LDL-cholesterol levels diagnostic of HoFH, less than half did not recognize the diagnosis of HoFH in their patients and had difficulty in distinguishing HoFH from heterozygous familial hypercholesterolemia. This distinction is critical since patients with HoFH are at much higher risk of early and aggressive cardiovascular disease and warrant more intensive therapies. Historically, HoFH was diagnosed at LDL-cholesterol levels > 500 mg/dL; however, almost half of genetically proven homozygotes have untreated LDL-C levels < 500 mg/dL.^2^ These findings do not appear to have made it into general practice.

When assessing cardiovascular risk in a patient with HoFH, most clinicians would use a risk calculator. It is important to note that past and current cholesterol guidelines do not recommend using risk calculators in patients with HoFH and recommend treatment with high-intensity statins as soon as the clinical diagnosis is made.^1^ Most clinicians in our survey would prescribe high-intensity statins to treat HoFH patients. However, diet and exercise were chosen by many respondents as their first and second choices of therapy. They are important parts of the treatment of HoFH patients but have a relatively small effect. Only 24% of clinicians would start a male patient at age < 18 and only 20% a female patient at age < 18 on LDL-cholesterol lowering medication. Because the presence of severely elevated LDL-C from birth confers such a high risk of premature atherosclerosis, current guidelines for HoFH suggest starting medication at the time of diagnosis and several statins are approved for treating HoFH from age 10.^1^

In our survey, less than 2/3 of clinicians would use PCSK9 inhibitors and only 7% would use lomitapide in patients with HoFH, despite these drugs being approved to treat HoFH. Many would use fish oil or a fibrate which have little effect on LDL-cholesterol. About 2/3 of clinicians in our survey have access to a lipid specialist, but only 29% have access to an LDL apheresis center. Often patients with HoFH are difficult to treat and the lack of access to a lipid specialist and an apheresis center is a significant limitation to adequately treating patients with HoFH.

In conclusion, most primary care clinicians do not adequately recognize or treat HoFH, and do not have easy access to a lipid specialist or an apheresis center. Clinicians should suspect HoFH in any patient with a family history of high cholesterol or premature CV disease whose untreated LDL-cholesterol is > 400 mg/dL or whose treated level is > 300 mg/dL. Recent studies suggest that quick search terms in an electronic health record may improve the recognition of HoFH in clinical practice.^3^ There is a need for more education for primary care clinicians in recognizing and treating HoFH, and for greater access to lipid specialists and LDL-apheresis centers.
